# Positive Contrast MRI Techniques for Visualization of Iron-Loaded Hernia Mesh Implants in Patients

**DOI:** 10.1371/journal.pone.0155717

**Published:** 2016-05-18

**Authors:** Alexander Ciritsis, Daniel Truhn, Nienke L. Hansen, Jens Otto, Christiane K. Kuhl, Nils A. Kraemer

**Affiliations:** 1 Department of Diagnostic and Interventional Radiology, RWTH University Hospital Aachen, Aachen, Germany; 2 Department of General, Visceral and Transplant Surgery, RWTH University Hospital Aachen, Aachen, Germany; Le Fe Health Research Institute, SPAIN

## Abstract

**Object:**

In MRI, implants and devices can be delineated via susceptibility artefacts. To discriminate susceptibility voids from proton-free structures, different positive contrast techniques were implemented. The purpose of this study was to evaluate a pulse sequence-based positive contrast technique (PCSI) and a post-processing susceptibility gradient mapping algorithm (SGM) for visualization of iron loaded mesh implants in patients.

**Material and Methods:**

Five patients with iron-loaded MR-visible inguinal hernia mesh implants were examined at 1.5 Tesla. A gradient echo sequence (GRE; parameters: TR: 8.3ms; TE: 4.3ms; NSA:2; FA:20°; FOV:350mm²) and a PCSI sequence (parameters: TR: 25ms; TE: 4.6ms; NSA:4; FA:20°; FOV:350mm²) with on-resonant proton suppression were performed. SGM maps were calculated using two algorithms. Image quality and mesh delineation were independently evaluated by three radiologists.

**Results:**

On GRE, the iron-loaded meshes generated distinct susceptibility-induced signal voids. PCSI exhibited susceptibility differences including the meshes as hyperintense signals. SGM exhibited susceptibility differences with positive contrast. Visually, the different algorithms presented no significant differences. Overall, the diagnostic value was rated best in GRE whereas PCSI and SGM were barely “sufficient”.

**Conclusion:**

Both “positive contrast” techniques depicted implanted meshes with hyperintense signal. SGM comes without additional acquisition time and can therefore be utilized in every patient.

## Introduction

The MRI visualization of susceptibility differences is commonly based on the signal loss due to T2* shortening originating from local magnetic field alteration [1, 2].

These effects can be used to visualize intentionally induced susceptibility differences in devices such as stents, meshes for hernia repair or to track iron-oxide labelled stem cells.

In previous studies, a method has been successfully established to achieve complete visualization of iron-loaded mesh implants for hernia repair via susceptibility artefacts on gradient echo sequences (GRE) in phantoms [3], animals [4, 5] and patients [6]. Although a detailed depiction of the implants was possible, such passive visualization via artefacts does not allow one to discriminate between the underlying cause of the signal voids–a (susceptibility) artefact or a true lack of protons.

Therefore, methods were proposed to convert the susceptibility induced signal loss into “positive contrast”. Primarily, such techniques were developed for tracking labelled stem cells [7–9]. Today, there is a broad range of different imaging techniques for various applications, including device imaging [10].

In contrast to most positive contrast imaging techniques, which are based on dedicated pulse sequences, Dahnke and colleagues proposed a post-processing algorithm to calculate the influence of local magnetic fields based on conventional GRE sequences–susceptibility gradient mapping (SGM) [11, 12]. Encouraged by positive results in animals (rabbits) [5] the purpose of this investigation was to evaluate a pulse sequence-based positive contrast technique and the post-processing algorithm SGM for visualization of iron loaded mesh implants in patients, and to assess their potential value in comparison to conventional MRI.

## Materials and Methods

### Patients

This study was approved by the local ethics committee at the RWTH Aachen Faculty of Medicine (code no. 194/11), and all patients provided written informed consent. Five patients (5 men; median age 57 years; range 39–76) suffering from inguinal hernia were laparoscopically treated using MR-visible mesh implants. One patient was treated on both sides, resulting in a total of n = 6 iron-loaded MR-visible mesh implants (DynaMesh ENDOLAP ^®^ visible, FEG Textiltechnik, Aachen, Germany).

### Magnetic Resonance Imaging

MRI examinations were performed on a clinical 1.5 Tesla scanner (Achieva, Philips Healthcare, Best, The Netherlands) using a 16-channel torso receiver coil (Sense XL Torso Coil, Philips Healthcare, Best, The Netherlands) one day after surgery. The MR sequence protocol included a conventional gradient echo sequence (GRE) and a positive contrast susceptibility imaging (PCSI) sequence.

The GRE was based on sequences that were previously used, in which the iron loaded implant was exhibited as distinct signal voids a homogenously hyperintense surrounding anatomy [4, 6]. The GRE sequence parameters are given in Table 1. Image data were acquired as modulus, real, imaginary, and phase images. Based on these, the susceptibility maps were subsequently calculated.

**Table 1:** Sequence parameters.

	Repetition time (TR)	Echo time (TE)	Number of signal averages	Flip angle (FA)	Fied of view (FOV)	Voxel size	Slice thickness	Scan duration
**GRE**	8.3 ms	4.3 ms	2	20°	350 mm²	0.95 x 0.97	5 mm	2 min 26 sec
**PCSI**	25 ms	4.6 ms	4	20°	350 mm²	0.95 x 0.97	5 mm	4min 52 sec

In previous animal studies [3, 4] positive contrast was achieved using the idea of Stuber. suppressing the on-resonant protons [13]. The PCSI pulse sequence was based on a slice selective gradient echo sequence with a broad pre-pulse of 120° flip angle and a duration of 3 milliseconds and without frequency offset.

### Post Processing

The susceptibility gradients for each voxel were calculated with the Philips Research Imaging Development Environment (PRIDE) (Philips Healthcare, Best, The Netherlands). Based on the GRE images, including phase data, the SGM algorithm assesses the echo shifting caused by local gradients for each voxel. The echo shifting is the result of interference between the read-out gradient and the z- component of local magnetic gradients [11, 12]:

$$m=-\frac{G_{sus}TE}{(G_{imaging}+G_{sus})\tau }$$ (1)

Here, *m* is the echo shift in K-space, *G*_*immaging*_ is the readout gradient, *G*_*sus*_ is the induced susceptibility gradient and *τ* is the inverse of the sampling rate. This relation also holds for echo-shifting in the phase-encoding and slice selection direction [14, 15].

Using the SGM-application PRIDE, two different algorithms were applied; Short Term Fourier Transform (STFT) and the True Resolution (TrueRes) algorithm [11, 12, 16].

The STFT approach is based on a multiplication of the image matrix with a rectangular window function. After transforming this into the Fourier domain the echo shift is determined. This process is repeated on a pixel-by-pixel basis in all available image dimensions. creating a new image stack based on echo shift values [11].

By truncating k-space, the TrueRes method determines the k-space line where a sudden change in pixel intensity occurs. The echo shift can be assessed by noting this point in k-space [12, 16].

### Image evaluation

Three clinical radiologists independently scored all images (GRE, PCSI and SGM) on a 4-point scale (1: “insufficient for diagnosis”, 2: “barely sufficient for diagnosis”, 3: “sufficient for diagnosis”, 4: “optimal for diagnosis”) with respect to the following criteria: (1) visibility of the mesh, (2) differentiation from other hypo- or hyperintense structures (3) overall diagnostic value. No quantitative analysis was performed as measuring signal- or contrast-to-noise ratios do not reflect the radiologist’s ability to make a diagnosis (Table 2).

**Table 2:** Results of the image evaluation.

Criterion	Visual conspicuity of the mesh				Differentiation from other structures				Overall diagnostic value to assess mesh structure			
Modality	*GRE*	*PCSI*	*STFT*	*TrueRes*	*GRE*	*PCSI*	*STFT*	*TrueRes*	*GRE*	*PCSI*	*STFT*	*TrueRes*
**Mean Value**	3.9	1.9	3.5	3.0	3.0	1.8	2.7	2.3	3.9	1.9	3.5	3.0
**Median**	4	2	4	3	3	2	3	2	3	1	3	2
**Standard deviation**	0.4	0.9	0.6	0.4	0.9	0.8	0.8	0.7	0.4	0.9	0.6	0.9

## Results

GRE and PCSI images were successfully acquired and susceptibility gradient maps were generated in all patients.

**Fig 1:**
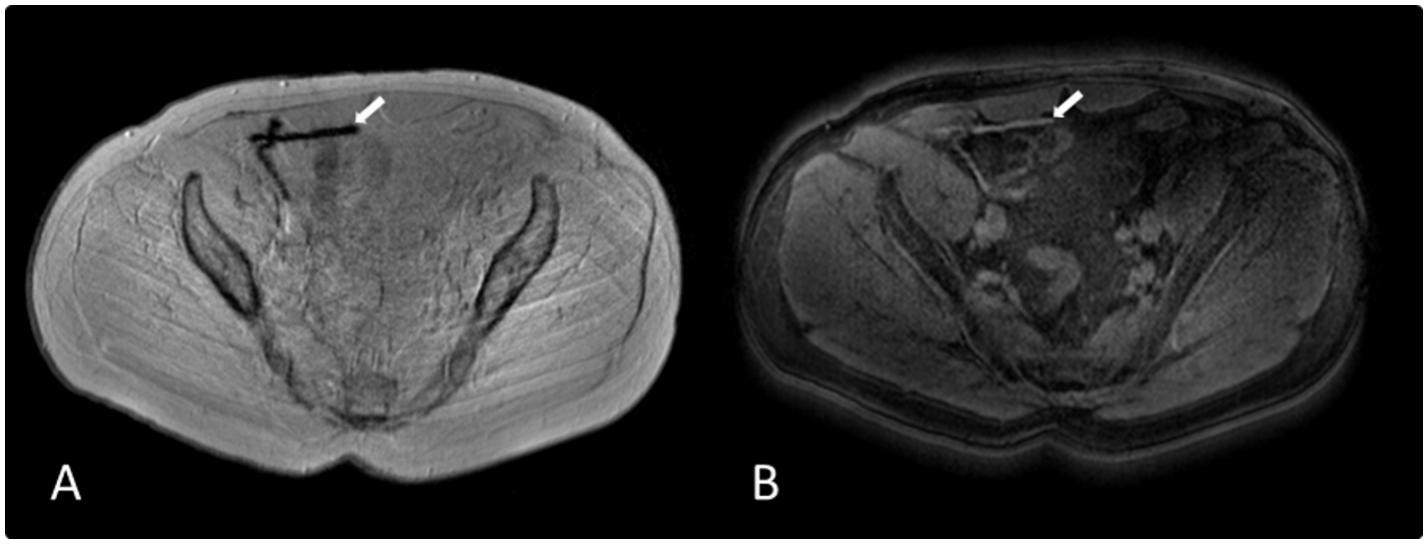
Transversely orientated MR images of a patient’s groin after laparoscopic hernia repair using a mesh implant (arrows). A: On GRE, the mesh-induced signal voids (arrow) clearly contrast to the surrounding hyperintense anatomy resulting in precise delineation. B: On PCSI, the susceptibility differences induced by the iron-loaded mesh implant (arrow) exhibit as hyperintense signals.

On GRE, the iron-loaded mesh implants generated distinct susceptibility-induced signal voids (Fig 1) and rendered the mesh; average conspicuity was rated as “optimal” (3.9+/-0.4). Differentiation from signal voids caused by other structures, e.g. intra- or extraluminal air, was rated as being “sufficient” (3.0+/-0.8). The overall diagnostic value of GRE to assess the mesh implant was rated “sufficient” or”optimal” (3.4+-0.5).

**Fig 2:**
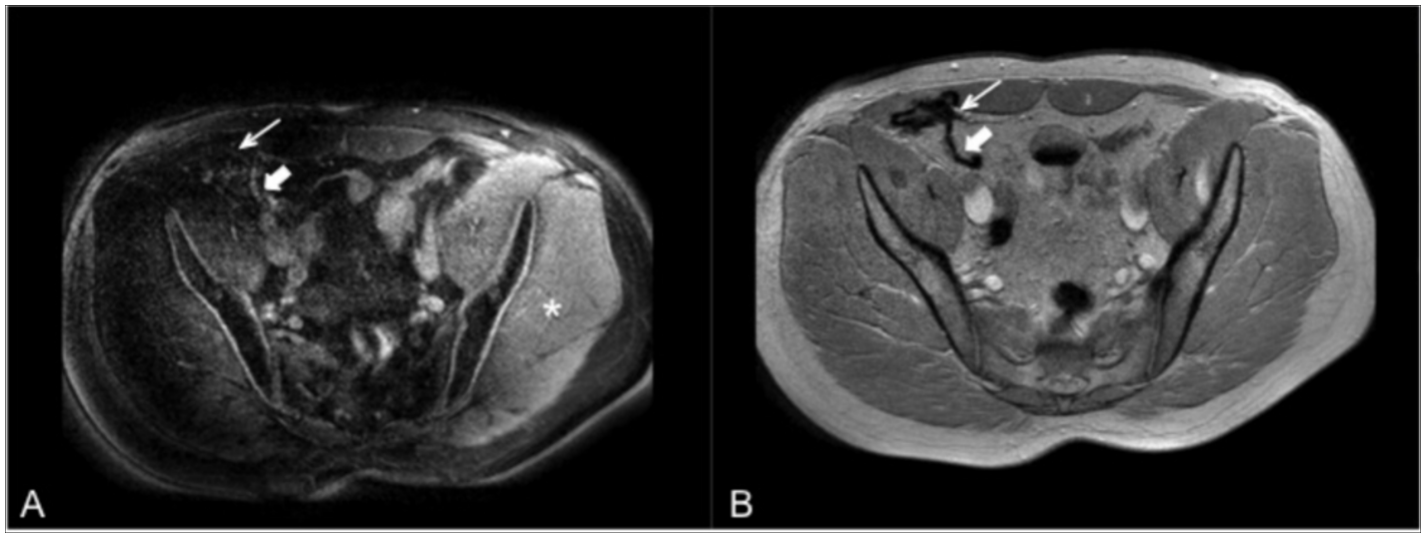
Transversely orientated MR images with a heavily folded mesh configuration (pronounced in the ventral mesh parts). On PCSI (A) the mesh can be discerned when configurated even (thick arrow), but fails to exhibit positive contrast when heavily folded (slim arrow). On GRE images (B), the mesh can be depicted as signal voids irrespective of its configuration (thick/slim arrows). On PCSI (A), B1-inhomogeneties result in inhomogenous background suppression (asterisk, *). On GRE (B), this effect is not present.

PCSI displays susceptibility differences as hyperintense signals. The iron-loaded mesh implants also appears hyperintense (Fig 1). Visibility of the meshes and their differentiation from other structures were rated as being “barely sufficient” (1.9+/-0.9 and 1.8+/-0.8, respectively). For these two scores, the between-patient variability was broad, with some meshes clearly visible (Fig 1), and others barely/not visible (Fig 2). The average overall diagnostic value was rated as “insufficient” (1.6+/- 0.7).

Susceptibility gradient maps were successfully calculated using both algorithms (STFT and TrueRes).

**Fig 3:**
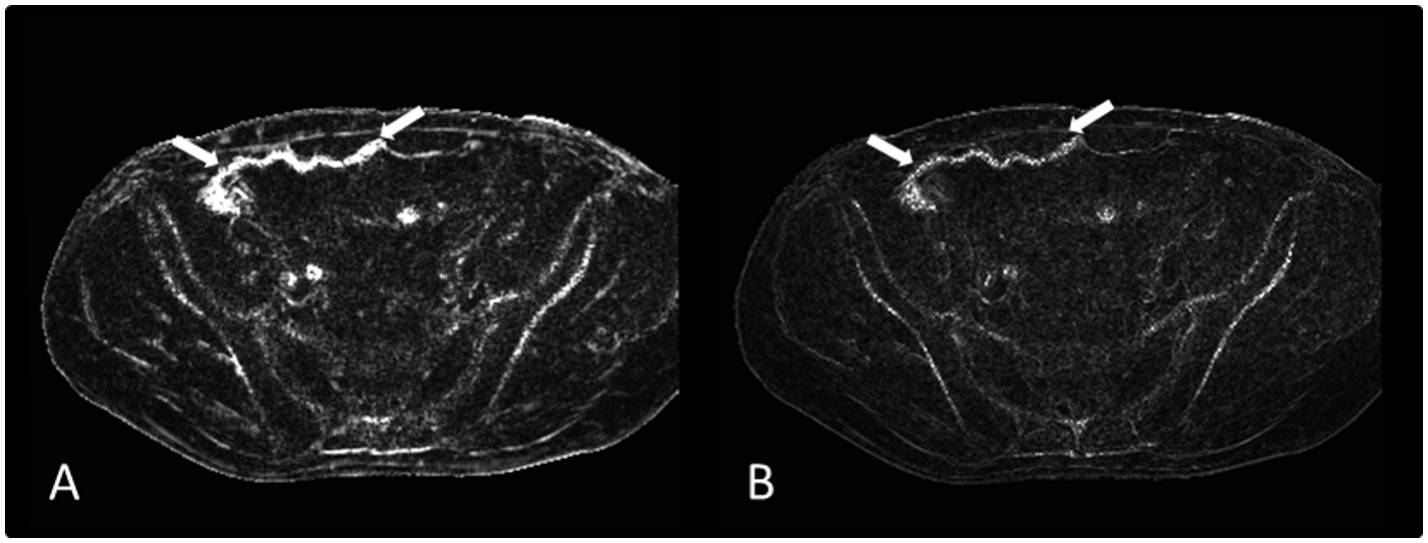
Based on GRE data, susceptibility gradient maps (SGM) were reconstructed by using two different algorithms: A: the Short Term Fourier Transform (STFT) and B: the True Resolution (TrueRes) algorithm. A significant difference regarding the visibility of the mesh implant (arrows) was not found.

Visually, the maps generated with these two algorithms presented no significant differences (Fig 3). In the ratings, no significant differences were found.

Both SGM algorithms exhibited the susceptibility differences with positive contrast.

The visibility of meshes by both methods was rated as “sufficient” to “optimal” (STFT: 3.5 +/-0.6; TrueRes: 3.0 +/- 0.9). The scores for differentiation of meshes from other structures ranged between “barely sufficient” and “sufficient” (STFT: 2.7 +/- 0.8; TrueRes: 2.3 +/- 0.7). Also, overall diagnostic value was rated as “barely sufficient” or “sufficient”, with scores ranging from: STFT: 2.8 +/- 0.9; TrueRes: 2.3 +/- 0.9).

The two algorithms to generate SG-maps (STFT and TrueRes) received almost identical scores in all cases. No significant differences were found between the two algorithms.

## Discussion

This patient study presents two positive contrast MRI techniques that can be used to image iron-loaded polymer-based mesh implants with positive MR contrast.

Passive visualization via signal voids due to susceptibility artefacts is the most common method to visualize metallic devices or labelled implants [17, 18] Gradient echo sequences are most susceptible for low T2* values that occur in the immediate vicinity due to strong local magnetic gradients.

As the T2* effects on gradient echo sequences are highly reproducible, passive visualization is very robust. Moreover, a wide set of gradient echo sequences for various clinical applications are available and well established. Last but not least, radiologists are used to these images and their contrasts; so susceptibility artefacts can be identified easily by a radiologist.

However, on gradient echo sequences, signal voids caused by susceptibility differences cannot be differentiated from signal voids due to other reasons such as bowel air, or materials with extremely short T2 values (e.g. cortical bone, or implanted plastic material). Rationale for the development of positive contrast techniques such as PCSI and SGM is to enable the differentiation between these different causes of signal voids.

In previous phantom and small animal studies, both techniques worked well [3, 10]. In our experience, this held also true for experiments in larger animals (domestic pigs). Accordingly, aim of this study was to investigate the utility of PCSI and SGM in patients.

PCSI requires an accurate on-resonant signal suppression as well as a reliable signal depiction. The duration of the suppression pulse defines the bandwidth of the suppressed spectrum, in this case about 350 Hz. Originally, a fat-suppressed inversion recovery spin echo sequence with one selective pre-pulse to suppress the on-resonant water protons had been proposed [13,19]. In subsequent studies, this on-resonant signal suppression was applied by gradient echo sequences using two spectral pre-pulses [10, 20]. In our study, a slice selective gradient echo sequence was adapted to account for difficulties of human in vivo imaging such as motion.

Difficulties with PCSI may arise due to patient motion, which leads to artefacts and blurring of the subtle signal acquired by conventional PCSI sequences. As breath triggering resulted in excessively long acquisition times, the sequences were adapted such that on-resonant protons are suppressed for low k-space values and high k-space values are sampled while the signal of on-resonant spins is returning. Rationale is that positive contrast by off-resonant protons can be preserved while high k-space values can both be sampled quickly and with sufficient signal. This leads to a pulse sequence that is more robust with regards to motion.

In spite of these technical adjustments, in this study, PCSI did not prove to be suitable for a detailed clinical evaluation of mesh implants. This result is not in keeping with our prior experiences of PCSI in small and large animals. Several different explanations are conceivable for this discrepancy:

PSCI is a very motion-sensitive technique. At the same time, PCSI requires long acquisition times because the number of off-resonant protons is low, such that multiple signal averages (NSA = 4) are necessary. All of our large animal experiments had been conducted with the animal in general anaesthesia, with the animal intubated and ventilated. It is then easy to obtain breath-hold sequences because ventilation is only re-started once image acquisition is completed. As opposed to this, breath hold pulse sequences are more challenging in clinical patients because they would be associated with prohibitively long breath hold times. Accordingly, for this study, PCSI had to be acquired with the patient free breathing. This, however, led to substantial image degradation due to motion artefacts.

A good on-resonant signal suppression also relies on a homogenous pulse application. Yet B1-inomogeneties are more pronounced in patients than in large animals, such that homogenous suppression proved to be more challenging. In some cases, this led to variations in contrast (Fig 2) limiting mesh visibility and overall diagnostic value.

Last, in our animal studies, the meshes had been implanted in a configuration that exhibited only few folds, probably because meshes had been implanted via open surgery. In patients, meshes had been implanted through laparoscopic surgery which, possibly due to post-operative release of pneumo-peritoneum, led to stronger folding of the mesh implant. Due to these folds, the amount of iron particles per voxel is increased, T2* decreases, and the off-resonance spectrum becomes even broader. As the measuring bandwidth per voxel is limited, the signal is spread out over multiple pixels in frequency encoding direction–yielding a signal decrease. This is supported by our observation that positive contrast was best in the unfolded, even parts of the mesh (Fig 2). The locally variable amount of iron in the folded meshes led to the fact that in PCSI images, it was difficult to delineate the mesh implants in their entirety. This led to significantly lower scores for PCSI compared with SGM and “conventional” GRE imaging at clinical image interpretation.

All these factors resulted in an inconsistent image quality of PCSI. Thus, PCSI appears to be not suitable for clinical imaging of iron-loaded implants.

Moreover, severe post-surgical changes such as edema, hematoma, and air bubbles might comprise the post-surgical implant visualization in any imaging technique. In this study, the amount of post-surgical changes found on conventional imaging were only subtle and were consequently not taken into account regarding the image evaluation.

For SGM, positive contrast from susceptibility differences can be achieved via post-processing of conventional gradient echo sequences. These sequences are established, robust and broadly available. Both SGM algorithms evaluated by us, i.e. TrueRes and STFT, exhibited the mesh implant with positive contrast, enabling a clear-cut distinction of the mesh from surrounding tissue such as muscle and fat. However, a distinction from signal free structures was not feasible. Both algorithms use the phase information to calculate SGM. In cases of signal voids, the SGM calculation is performed with the arbitrary phase information given from noise, resulting in random values including “positive contrast”. This is why not only the high SGM values in/at the mesh, but also air filled bowel and cortical bone appeared bright, i.e. with positive contrast. Accordingly, this could result in impaired discrimination between air and susceptibility induced voids, the primary goal of any positive contrast technique. Still, as SGM post-processing does not require additional scan time and can be performed with pulse sequences which are needed for successful mesh visualization anyway [6], it seems to be an easy method to achieve potentially helpful information. Moreover, susceptibility maps quantify the amount of susceptibility difference and thus provide additional potentially useful quantitative information about the observed device.

Further improvement of the post-processing algorithm might also improve the differentiation of susceptibility induced voids from proton free structures and could also provide more detailed information on susceptibility gradients [21].

## Conclusion

In this patient study, both “positive contrast” techniques, PCSI and SGM, were successfully used to depict magnetically labelled implanted meshes with hyperintense signal. However, clinical image evaluation revealed that both methods do not provide satisfactory mesh visibility, and differentiation of the mesh from other low-signal structures. Accordingly, conventional pulse sequences are needed for evaluation of iron loaded mesh implants. Yet if conventional gradient echo sequences are acquired, SGM comes without additional acquisition time and can therefore be utilized in every patient. Current research in new post-processing algorithms for quantitative susceptibility mapping might further improve the delineation of iron-labelled implants and devices.

## Supporting Information

S1 Table Image evaluation. (DOCX)
