# Correction: Positive Contrast MRI Techniques for Visualization of Iron-Loaded Hernia Mesh Implants in Patients

**DOI:** 10.1371/journal.pone.0163630

**Published:** 2016-09-20

**Authors:** Alexander Ciritsis, Daniel Truhn, Nienke L. Hansen, Jens Otto, Christiane K. Kuhl, Nils A. Kraemer

There are errors in the Author Contributions. The correct contributions are: Wrote the Paper: AC. Analyzed the data: AC NK. Contributed methodology: AC NK. Contributed investigation: DT NH. Reviewed the paper: NK CK. Contributed resources: CK. Contributed supervision: NK. Contributed as surgeon: JO

## References

[1] Ciritsis A, Truhn D, Hansen NL, Otto J, Kuhl CK, Kraemer NA (2016) Positive Contrast MRI Techniques for Visualization of Iron-Loaded Hernia Mesh Implants in Patients. PLoS ONE 11(5): e0155717 doi: 10.1371/journal.pone.0155717 27192201 10.1371/journal.pone.0155717 PMC4871409

