# Supplementary material for: Positive Contrast MRI Techniques for Visualization of Iron-Loaded Hernia Mesh Implants in Patients
Source: PLoS One. 2016 May 18;11(5):e0155717. doi: 10.1371/journal.pone.0155717 (PMC4871409; doi:10.1371/journal.pone.0155717)
Supplement: S1 Table — (DOCX) [file pone.0155717.s001.docx]

**Visual conspicuity oft he mesh**

|  |  | **Visual Conspicuity of mesh** | | | |
| --- | --- | --- | --- | --- | --- |
|  |  | **STFT** | **TrueRes** | **GRE** | **PCSI** |
| *Observer 1* | *Patient 1* | 4 | 3 | 4 | 3 |
|  | *Patient 2* | 3 | 4 | 4 | 1 |
|  | *Patient 3* | 4 | 4 | 4 | 2 |
|  | *Patient 4* | 3 | 3 | 4 | 1 |
|  | *Patient 5* | 3 | 3 | 4 | 3 |
|  |  |  |  |  |  |
| *Observer 2* | *Patient 1* | 3 | 3 | 4 | 3 |
|  | *Patient 2* | 3 | 3 | 4 | 1 |
|  | *Patient 3* | 4 | 3 | 4 | 2 |
|  | *Patient 4* | 3 | 4 | 4 | 2 |
|  | *Patient 5* | 4 | 4 | 4 | 3 |
|  |  |  |  |  |  |
| *Observer 3* | *Patient 1* | 2 | 1 | 4 | 3 |
|  | *Patient 2* | 4 | 2 | 4 | 1 |
|  | *Patient 3* | 4 | 2 | 3 | 2 |
|  | *Patient 4* | 4 | 3 | 3 | 1 |
|  | *Patient 5* | 4 | 3 | 4 | 1 |
|  |  |  |  |  |  |
| Mean value |  | 3,47 | 3,00 | 3,87 | 1,93 |
| Median |  | 4 | 3 | 4 | 2 |
| Standart deviation |  | 0,64 | 0,85 | 0,35 | 0,88 |

**Differentiation from other structures**

|  |  | **Differentiation from other structures** | | | |
| --- | --- | --- | --- | --- | --- |
|  |  | **STFT** | **TrueRes** | **GRE** | **PCSI** |
| *Observer 1* | *Patient 1* | 4 | 3 | 4 | 3 |
|  | *Patient 2* | 3 | 3 | 3 | 1 |
|  | *Patient 3* | 2 | 2 | 3 | 2 |
|  | *Patient 4* | 3 | 3 | 3 | 2 |
|  | *Patient 5* | 3 | 3 | 3 | 3 |
|  |  |  |  |  |  |
| *Observer 2* | *Patient 1* | 2 | 2 | 3 | 3 |
|  | *Patient 2* | 2 | 2 | 3 | 1 |
|  | *Patient 3* | 3 | 3 | 4 | 2 |
|  | *Patient 4* | 3 | 3 | 3 | 2 |
|  | *Patient 5* | 4 | 3 | 4 | 2 |
|  |  |  |  |  |  |
| *Observer 3* | *Patient 1* | 1 | 1 | 2 | 2 |
|  | *Patient 2* | 2 | 1 | 1 | 1 |
|  | *Patient 3* | 3 | 2 | 3 | 1 |
|  | *Patient 4* | 2 | 2 | 2 | 1 |
|  | *Patient 5* | 3 | 2 | 4 | 1 |
|  |  |  |  |  |  |
| Mean value |  | 2,67 | 2,33 | 3,00 | 1,80 |
| Median |  | 3 | 2 | 3 | 2 |
| Standart deviation |  | 0,82 | 0,72 | 0,85 | 0,77 |

**Overall diagnostic value to assess mesh structure**

|  |  | **Overall diagnostic value to assess mesh structure** | | | |
| --- | --- | --- | --- | --- | --- |
|  |  | **STFT** | **TrueRes** | **GRE** | **PCSI** |
| *Observer 1* | *Patient 1* | 4 | 4 | 4 | 2 |
|  | *Patient 2* | 3 | 3 | 3 | 1 |
|  | *Patient 3* | 2 | 2 | 3 | 2 |
|  | *Patient 4* | 2 | 2 | 3 | 1 |
|  | *Patient 5* | 2 | 2 | 3 | 2 |
|  |  |  |  |  |  |
| *Observer 2* | *Patient 1* | 2 | 2 | 3 | 3 |
|  | *Patient 2* | 2 | 2 | 3 | 1 |
|  | *Patient 3* | 3 | 3 | 4 | 1 |
|  | *Patient 4* | 4 | 4 | 4 | 2 |
|  | *Patient 5* | 4 | 3 | 4 | 2 |
|  |  |  |  |  |  |
| *Observer 3* | *Patient 1* | 2 | 1 | 3 | 3 |
|  | *Patient 2* | 3 | 2 | 4 | 1 |
|  | *Patient 3* | 3 | 1 | 3 | 1 |
|  | *Patient 4* | 2 | 2 | 3 | 1 |
|  | *Patient 5* | 4 | 2 | 4 | 1 |
|  |  |  |  |  |  |
| Mean value |  | 2,80 | 2,33 | 3,40 | 1,60 |
| Median |  | 3 | 2 | 3 | 1 |
| Standart deviation |  | 0,86 | 0,90 | 0,51 | 0,74 |

**MRI - sequence parameters**

|  | **Repetition time**  **(TR)** | **Echo time**  **(TE)** | **Number of signal averages**  **(NSA)** | **Turbo spin echo factor (TSE)** | **Flip angle**  **(FA)** | **Filed of view**  **(FOV)** | **Voxel size** | **Slice thickness** | **Scan duration** |
| --- | --- | --- | --- | --- | --- | --- | --- | --- | --- |
| **GRE** | 8.3 ms | 4.3 ms | 2 | 10 | 20° | 350 mm² | 0.95 x 0.97 | 5 mm | 2 min 26 sec |
|  |  |  |  |  |  |  |  |  |  |
| **PCSI** | 25 ms | 4.6 ms | 4 | 108 | 20° | 350 mm² | 0.95 x 0.97 | 5 mm |  |

**Graphical abstract**

Two different approaches of positive contrast imaging (one direct sequence based technique and two post-processing algorithms), were successfully applied for in patient visualization of hernia mesh implants.

**Positive Contrast Techniques For Visualization Of Iron-Loaded Mesh Implants In Patients**


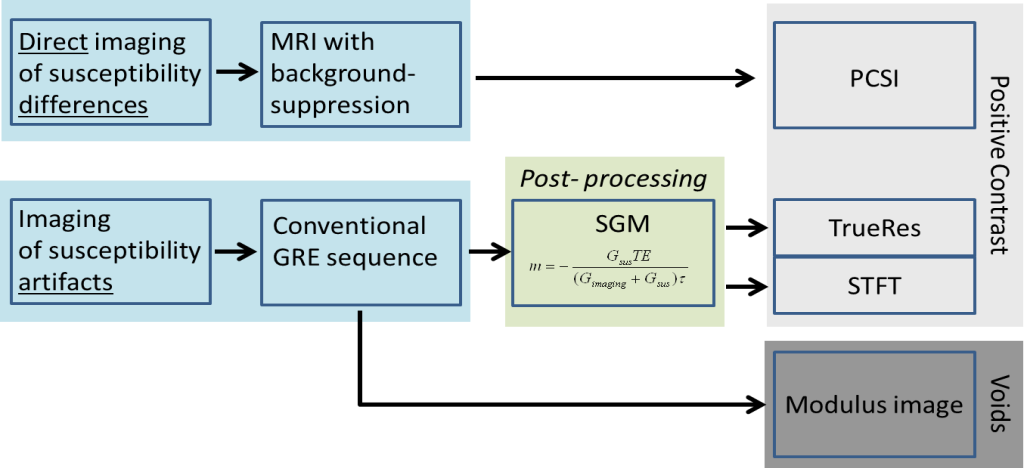


Alexander Ciritsis, Daniel Truhn, Nienke L. Hansen, Jens Otto, Christiane K. Kuhl, and Nils A. Kraemer
